# “Mom Said after the Spring Festival, I’ve Grown a Year”: Chinese Preschoolers’ Perspectives on Growing Up

**DOI:** 10.3390/bs14030253

**Published:** 2024-03-20

**Authors:** Yinshan Su, Jin Huang

**Affiliations:** School of Education Science, Nanjing Normal University, Nanjing 210046, China; 190601016@nnu.edu.cn

**Keywords:** preschool children, growing up, children’s perspectives, Merleau-Ponty’s phenomenology

## Abstract

Previous studies on child development have emphasized universal developmental stages and socialization, overlooking a direct investigation of young children’s subjective understanding of growing up. This study explored the perspectives of preschool children on growing up. Participant observations, semi-structured interviews, and drawing-telling were employed to investigate 56 urban Chinese preschoolers. The theoretical framework adopted for this study was Merleau-Ponty’s existential phenomenology, providing a lens through which the children’s voices were elucidated. The results revealed that children perceive their growth holistically across four themes: body, space, skills, and relations. Their perception of growing up adheres to a structure–agency duality, where social influences and children’s agency coalesce to shape their understanding of growing up. Adults contribute by embedding significance in daily situations and designated “occupations”, while children actively reinterpret these societal narratives, forging their conceptions of growing up. These findings suggest a need for educational approaches that resonate with children’s interpretations of their evolving lifeworld beyond merely imparting knowledge.

## 1. Introduction

Scientists focus on changes in matter, farmers pay attention to changes in crops, and educators concentrate on changes and development in children. Whether in everyday life or children-related academic knowledge, people consider growth a pivotal theme in early childhood [1]. Scholars from various disciplines approach children’s developmental issues through different lenses, with psychology and sociology being two major mainstream disciplines [2]. Traditional developmental psychologists employ experimental methods and other quantitative approaches to identify the developmental stages in aspects such as children’s cognitive, language, moral, and socio-emotional development. This involves examining the transitions between developmental stages and psychological constructs detached from the socio-cultural context they inhabited [3,4,5]. Socialization theory explores how children, within institutions such as schools and families, are socialized from their initial biological state into individuals who conform to societal expectations [2]. Anthropologists study different ethnotheories in parenting practices and the resulting behaviors of children across cultures [6,7,8], viewing children’s behaviors as direct responses to external stimuli like rewards and punishments [9]. These studies adopt an external perspective to explore children’s development, creating a certain distance from the children’s viewpoints on their specific growth. Additionally, some phenomenological studies, influenced by existential philosophers such as Heidegger and Merleau-Ponty, explore the views of children as subjective beings regarding bodily changes [10], going to school [11], having pride [12], keeping secrets [13], childhood and adulthood [14,15]. However, there is no specialized research regarding young children’s perceptions of growing up.

There seems to be scholarly agreement that children are not “the adult-in-training” or “the miniature adult” [16], with lower rationality and less emotional control; rather, their consciousness differs from that of adults in terms of both content and organization [17]. Children, as stakeholders [18], have the right to express their thoughts on matters concerning them and to be acknowledged and documented [17,19,20]. Existing research also indicates that children’s voices can be used as genuine and valid evidence to enable adults to gain significant knowledge [21,22].

Therefore, this study aims to investigate the subjective, nuanced, and diverse understanding of growing up in the eyes of Chinese preschoolers. By doing this, educators can create an engaging and supportive learning environment that fosters their growth and is tailored to their individuality [17]. As children live in a socio-cultural context, their development and growth result from the interaction between individuals and society, preschool, and family [23,24]. We further explore how particular cultures influence preschoolers’ views on growing up.

## 2. Methods

### 2.1. Design

This study sought to comprehend and interpret the perspectives on growing up of young children, avoiding predefined hypotheses to instead formulate concepts and theories through continuous data analysis and comparison during the fieldwork. Hence, the study adopted a phenomenological methodology. This approach defends the significance of individuals’ lived experiences, aims to capture the richness of life, and brings up transformative thoughts on existence, thus allowing for a deeper appreciation of the children’s subjective views. Van Manen highlights that phenomenology in pedagogy brings about a theoretical and practical focus on the lived worlds of children and educators, as experienced in everyday situations and relations [25]. 

### 2.2. Research Participants

This study was conducted in three public urban preschools (for children aged three to six) in Nanjing City, Jiangsu Province. According to educational statistics, by the end of 2022, the Chinese urbanization rate of the permanent resident population reached 65.22% [26]. The three preschools were therefore selected to represent urban preschools in China. A “*daban*”, the senior class in Chinese preschool education, was selected for study in each preschool. In China, preschools have three different class levels: *xiaoban* for children aged 3–4, *zhongban* for children aged 4–5, and *daban* typically for children aged 5–6. *Daban* is the final stage before entering primary school, emphasizing preparation for formal education. *Daban* children were chosen because they had more life experiences than the younger children, they would have more ideas about their developmental changes, and their language would be more advanced [27]. One class of children was selected as the field in each preschool rather than a scattered group of children in different classes since the researcher needed to spend time with them to learn more about their life scenarios and backgrounds. Referring to the class teachers’ recommendations, we selected children with differentiated personalities and performances in class activities, and with an inclination to share with the researcher as participants [10]. The participants lending their voices to the research were 56 children (including 27 boys and 29 girls) aged 5 to 6 years eventually (see Table S1, Supplementary Materials).

The selection of three distinct preschools as the research sites for this study was an adaptive response to evolving circumstances rather than a predetermined research design. Initially, the research commenced at A Preschool, a partner institution of the researcher’s advisor, which granted access to its *daban*. As the study unfolded, an opportunity arose to gather more extensive data at B Preschool when planning a thematic activity titled “I Have Grown Up”. Considering the potential wealth of information, the researcher engaged with this new class in parallel from December 2020 to the children’s graduation in June 2021. However, due to COVID-19, the preschools implemented strict visitor controls, and classes were occasionally suspended, resulting in the researcher having less time than anticipated to collect data within the preschools. At that time, the researcher was concerned about the sufficiency of the collected data and attempted to re-enter the original sites in May 2022. However, the applications were hampered by changing visitor policies amidst the pandemic, consequently leading the researcher to seek the third preschool. C Preschool became accessible through an introduction letter from the researcher’s advisor. 

### 2.3. Data Collection and Procedure

Participant observations, semi-structured interviews, and drawing-telling were employed to collect data in this study.

This study began with participant observations, where the researcher disclosed their identities and stayed with the participants to capture the dynamics of situations, events, behaviors, relationships, roles and more [28]. Two weeks of a “familiarization” phase in the classroom was conducted [29]. The researcher took the “least-adult role”, explicitly rejecting any authority associated with being an adult among children or a researcher among the researched to interact with young children [30]. By doing so, the researcher gained the children’s trust, fostering a willingness and earnestness in them to contemplate and respond to the questions posed by the researcher. This, to a certain extent, ensured the reliability and validity of the data obtained from the children. In the formal observation phase, the researcher stayed in the preschools and meticulously documented children’s activities from 8 a.m. to 4 p.m. every Monday to Friday to understand the schedule and content of their activities and their interactions with teachers and peers within the natural preschool environment for six months.

Semi-structured interviews, as Wengraf notes, encompass two fundamental characteristics: firstly, the interview questions are partially prepared beforehand but still offer flexibility for both the interviewer and interviewee to explore topics; secondly, the interviews facilitate a thorough exploration of the details and complex processes behind the apparent facts [31,32]. The guiding questions for the children were as follows: “What makes you feel that you are growing up?” and “What have you noticed happening?”. The researcher also invited parents to provide photos or videos of their children, which recorded the children’s developmental changes. Then, these materials were used as stimuli in the interviews to awaken the children’s memories and elicit more expansive ideas. To avoid the preconceived effects of stimuli on the children’s self-perception, the researcher conducted the interviews without photos or videos and, at a later point, used these stimuli. When more background information was needed to understand a child’s narrative better, the child’s parents and teachers were also included in the interviews.

Drawing-telling leverages drawing as an expressive, and embodied medium to allow children to create narratives around their paintings. For young children who have yet to master reading and writing formally, drawing is one of their early literacies. Children are familiar with drawing and excel in these activities. An increasing number of studies incorporate them into the research methods. Researchers have found that utilizing such tools can enhance children’s memory retrieval, support their meaning-making, and enable them to express more of their ideas effectively [21,33,34,35,36]. The researcher, therefore, invited the children to draw what made them feel they were growing up and then narrate the stories behind their drawings, while not limiting the discussion to what is in the picture. 

This methodological triangulation reinforced the validity of the results through the convergence of multiple data sources. To prevent potential data loss during the data collection, the interviews and drawing-telling sessions were audio-recorded with the participants’ consent and transcribed verbatim in Word files.

### 2.4. Data Analysis

The analysis of the transcripts was a combination of theory-driven and data-driven. Merleau-Ponty’s existentials of the lifeworld, a term that refers to the experiential qualities of the lifeworld [37], served as an index for the analytical process but not a kind of “checklist” in advance of the things themselves, which would risk the phenomenological attitude [38]. The existentials, such as corporeality, spatiality, projects, relationality, and temporality, describe the way human beings experience the affairs of their day-to-day existence regardless of their historical, cultural, or social circumstances. Corporeality refers to the reality of our constant physical presence in the world, where we perceive the world and others in our lived body. Spatiality denotes the felt space we perceive and are influenced by. Unlike numerical space (length, height, and depth), it is more challenging to express in language [25]. Projects represent individuals carrying out certain activities they are committed to and regard as central to their lives [38]. Relationality is the lived relationship we maintain with others in the shared interpersonal world. These existentials can be differentiated but not separated [25,39].

Thematic analysis [25] was used to organize the collected data in data-driven stages. First, we performed a wholistic, selective, and detailed reading of the transcripts [25] , then we identified meaningful content as codes. When a child says, “I can dress myself, I am growing up”, the corresponding code is “dress independently”. Subsequently, related and linked codes were grouped into subcategories and categories. For example, “dress independently” and “brush teeth and wash face independently” are both classified into the subcategory of “self-care skills”, and the subcategories of “self-care skills” and “self-protection skills” are merged into the category of “daily living skills”. Finally, the categories were listed on a separate sheet to explore their correlations. Drawing on Merleau-Ponty’s theory, specific categories were merged to generate themes (see Table 1 below), establishing the content framework for the children’s perceptions of growing up. For instance, “daily living skills”, “athletic skills”, “artistic skills”, “recreational skills”, and “scholastic skills” are specific projects in children’s everyday life, so they were integrated as Theme Skills. It is important to note that “helping at home” involves some projects and skills as well; however, the researchers placed it under Theme Relations because children tend to perceive their chores more in terms of interpersonal relationships, as they may say, “I help someone with something” rather than emphasizing how well the chores are performed.

To ensure the reliability of the data analysis, each author initially approached the material independently. Then, the authors discussed it, eventually resulting in the coding system that everyone agreed on.

### 2.5. Ethical Considerations

Several ethical considerations were addressed in this study using the principles proposed by Hill [40] and Sanjari et al. [41]. Formal ethical approval was granted by the Institutional Review Board (IRB) of the researchers’ university. Both parents’ written informed and children’s oral consent were garnered. Obtaining consent was viewed as an active and ongoing process. Participants were given the right to refuse to answer any question and to opt out of the research in any research session [42,43]. The researcher would record the interviews with the consent of the participants. The researcher explained to the children: “I want to record because what you say is interesting and important, but I’m afraid I will not remember it. I want to listen to it again when I return to campus”. They were also informed that the data would be anonymous and confidential.

## 3. Results

Based on the data analysis, four main themes and twelve categories were identified. Below, we elaborate on these themes and categories, providing direct quotes from the participants to support and illustrate each theme and category.

### 3.1. Body

This theme was the most frequently mentioned by the preschoolers participating in the study, specifically including two categories: physical changes and age.

Regarding physical changes, the children often described the enlargement in their physique, including an increase in height, weight and strength, elongation of the limbs and upper body, larger feet, as well as the transition from “little teeth” to “big teeth” (indicating the replacement of deciduous teeth with permanent teeth). Notably, few children mentioned the emergence of fine hairs on their fingers. Among these, the increase in body height was the most common response. Chenchen, a boy, shared: “*When in xiaoban, I was only five years old, shorter by a head compared to [my current height]. Then, I grew taller, shorter by half a head compared to [my current height], and now I’m here [pointing to the current height].*” This aligns with previous findings that highlight the central role of body height in young children’s self-awareness [44,45], with preschoolers paying close attention to their height and increasing interest in “growing taller” and “growing up” [10,46]. Interestingly, individual children doubted the relationship between physical changes and growth, as Yuyu, a girl, elaborated: “*That’s very funny. When my dad wanted to hold me, he hugged me and said, ‘Oh, you’re heavy.’ Then he said I was a ‘big kid’ and ‘grown up,’ but I don’t know if it’s true. I think he may have been thinking it, but I don’t think it makes sense. Everyone is heavy, so if you were very chubby when you were a little kid, even if you grow so big, do you still feel like you have grown up? … As a very young kid, if you are plum and strong at age one, I feel like you should have grown up just a little bit.*” 

Children also reported the infrequent phenomenon of physical loss and related it to growth. They talked extensively about losing a tooth.

*Yueyue (girl): The tooth fell out, and I felt like I was gonna grow taller real soon.*

*Researcher: How did you feel when your tooth fell out?*

*Yueyue: I was brushing my teeth, and then it fell out. I cried immediately because I thought a bug had bitten it off. [Later, I found out] there were no bugs on top; it was quite clean. (...)*

*Researcher: Did your mom or dad say anything?*

*Yueyue: My mom really likes it when I lose my tooth. When she sees me lose a tooth, she gets excited and yells. Then I said, “I don’t want to lose teeth”, then my mom said, “Losing teeth is great. Losing teeth makes you grow taller”. Then I believed her and stopped crying.*

During the observation, the researcher witnessed a child losing a tooth and the reactions of the surrounding children. The children were having lunch at the time. A child suddenly lost a tooth. The other children gathered around to look, and upon seeing this, Hetao, a boy, raised his voice and said to the child who lost the tooth: *“Wow! Congratulations! Your tooth fell out, and you’re one year older now!”*

Some children observed that their faces and arms were less chubby compared to their infancy, noting a perceived decrease in cuteness. Xinxin, a girl, for instance, commented, “*When I was little, I was very cute, with a round face. My sister, dad, and mom all praised me, and my grandparents praised me for being cute, very cute, very cute. Now they don’t praise me anymore; now mom and dad are busy.*” Despite recognizing they are not as cute and not receiving the same praise as before, the children did not exhibit notable emotional distress over these changes. Their reflections on past physical attributes did not betray a longing for their earlier chubby forms; instead, they articulated these changes with a sense of objective observation. Xuxu, a boy, noted a decrease in his flexibility and exemplified, “*I used to be able to eat my feet, but now I can’t.*”

Moreover, age is perceived as a physical attribute or characteristic of growing up from the children’s perspectives [47]. The children gauged their age through celebratory events like birthdays and the Spring Festival (also known as Chinese New Year, which marks the beginning of a new year and the farewell to the old, according to the lunar calendar), which are imbued with the opportunity to gather with loved ones, enjoy favorite foods, and engage in enjoyable activities [48]. Dudu, a girl, remarked: “*Having a birthday means getting to eat tasty cake, and changing the age, like how I go from being one year old to two years old;” “It’s the year of the Rat, then I grew and grew, and then it became the year of the Ox, and that means I got one year older. (…) Mom told me it starts with the Rat and ends with the Pig [each year in the lunar calendar is represented by one of the twelve Chinese zodiac animals, like rat, ox, and tiger]”.*

### 3.2. Space

Space mainly referred to the changes in educational and accessible space related to the children’s growing up. Regarding educational space, the children believed that transitioning from home to preschool, from *xiaoban* to *zhongban*, from *zhongban* to *daban*, and from preschool to primary school were essential to growing up. Sining, a girl, said: “*I feel that going to preschool and elementary school makes us grow up. I didn’t go to preschool when I was little, but I started going when I grew up.*” Yiyi, a boy, noted, “*I feel like growing up means you have to move up to daban.*” Miaomiao, a girl, said: “*I have grown up. I used to be in xiaoban and zhongban, but now I’m in daban.*”

For accessible space, the children reported a tension between expanding and narrowing as they grew up. When toddlers can stand upright and begin walking, they continuously push the boundaries of the space they explore, higher and farther. The children found themselves enjoying greater accessibility in daily living spaces and gaining more access rights to public places, such as participating in playground activities that require a certain height. Hanghang, a boy, noted, “*I feel like I’m growing taller slowly. I used only to reach the 20th floor [elevator floor indicator], but now I can reach the card swipe area... I can even reach the 32nd floor now.*” Lulu, a girl, said, “*Growing taller is great. If you’re tall, you can play a lot of fun things. Some things have height requirements, and if you don’t meet them, you can’t play. For example, I once went to a high-altitude rope course, which required a certain height, and since I didn’t meet it, I couldn’t play.*” 

Meanwhile, the constraints imposed by adults excluded the children from the space they used to use, such as not being allowed to jump on the sofa. They could play, explore, and engage in various activities when they were younger. Moreover, the frequency of outings with parents decreased as they enrolled in more extracurricular classes. Taking Lulu as an example again:

*Lulu: When I was little, I used to play a lot and didn’t have to do homework. I could watch TV freely and bounce on the sofa anytime. Now, Dad doesn’t allow that. When I was little, I could sneak into the kitchen to eat things, but now, Mom doesn’t let me. When I grow up, no food is left in the kitchen.*

*Researcher: What does your mom say when she doesn’t allow you to eat in the kitchen?*

*Lulu: “No, no, quickly go away. Go do your homework; don’t bother with my stuff”. ... I want to grow up quickly. Because when I grow up, I’ll have my power.*

*Researcher: What does “power” mean?*

*Lulu: It means I must do what Mom tells me to do now. However, when I grow up, I can do things I want to do, like eating ice cream.*

### 3.3. Skills

**Figure 1:**
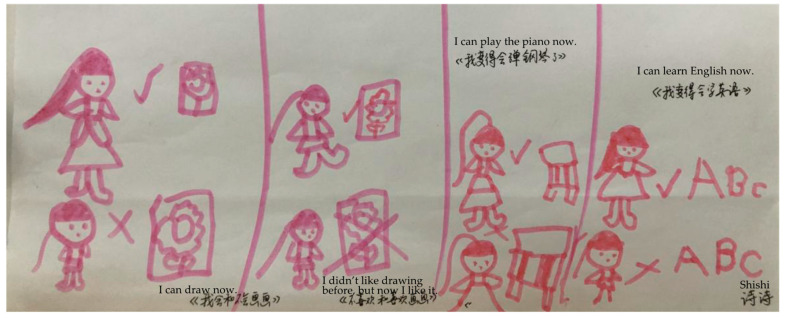
Shishi’s drawing: “I can draw now. I didn’t like drawing before, but now I like it. I can play the piano now. I can learn English now”.

The children believed that mastery and improvements in skills were indicative of their growing up. For instance, in Figure 1, Shishi, a girl, drew about her progress in drawing, piano playing, and English language learning, which made her feel like she was growing up. An analysis of data from all the participants, including Shishi, revealed that skills associated with growing up included daily living, athletic, artistic, recreational, and scholastic skills.

The daily living skills included basic self-care skills such as talking, walking, dressing and undressing, handling utensils and eating, spitting out fish bones, falling asleep, and brushing their teeth independently. For example, Momo, a girl, said, “*When I was little, I couldn’t turn up this collar [pointing to her top collar with fingers], but now I can.*” In addition, the children also linked growing up with learning self-protection skills in their daily lives, such as avoiding playing with fire and electricity, getting finger injuries when opening or closing doors and tossing water bottles. Haohao, a boy, said, “*When I was little, I played with fire, but not anymore. I also used to play with the gas stove when I was little.*”

The athletic skills referred to moderate to intense physical activities that engage various bodily functions, boasting a metabolic rate surpassing that of restful states [49]. The activities the children mentioned included, but were not limited to, playing basketball, shooting hoops, refining jump rope techniques from single jumps to continuous skipping, engaging in badminton, enhancing soccer ball kicking distance, acquiring swimming skills, increasing running velocity, and cycling, with or without the aid of training wheels, across a variety of common sports. Chengcheng, a boy, articulated his improvement in jumping rope, stating, “*I am better at jumping rope than before. Faster speed and more jumps, I can even hear the rope slicing through the air.*” Others reported mastering new pursuits, such as rollerblading and skateboarding, alongside traditional Chinese *kung fu*.

The artistic skills included drawing, dancing, and playing music. The children believed that growing up meant imitating and acquiring new fundamental techniques, including depicting overlapping relationships, coordinating colors, performing splits, bending backward, and playing music from scores. Art was associated with greater freedom, so the children also talked about progress in their thinking and creativity, as reflected in their ability to draw on their previous experiences and imagination to conceive and create artworks rather than imitate and copy model paintings or peers’ works. Yuyu explained, “*Before, I just copied from others when I drew. Now it’s better because I got some ideas when I look at the others’ drawings. I create my ideas by looking at nature or thinking about cartoons.*”

The recreational skills included advancing from simple to complex block constructions, engaging in more sophisticated games, and initiating programming activities. Zimo, a boy, noted a significant shift from playing with basic toy cars to tackling complex maze games, stating, “*When I was little, I could only play with baby toy cars. Now I’ve grown up, I can play advanced maze games.*”

The scholastic skills referred to knowledge and studying, including taking after-preschool academic classes for the school age in counting, pinyin, Chinese characters, and English, and achieving good grades in the exams of extracurricular courses. Xiaoquan, a boy, elaborated, “*When I was in xiaoban, I didn’t even know what eight plus eight equaled. Do you know? When I was little, I had to borrow my mom’s fingers to count eight plus eight. And then, for adding one thousand and two hundred, I had to borrow my mom’s fingers and toes to use, and my dad’s, sister’s, and aunt’s fingers and toes to use ... (Now) I know to take out a five from one eight, take out another five from another eight, add them together to get ten, and then the remaining two threes add up to six. Six plus ten equals sixteen.*” Grasping underlying principles can give children the joy of discovery, conquest, and a sense of efficacy, which motivates further learning [50]. This study also revealed the children’s negative emotions about excessive and mandatory homework. As Xinxin expressed, “*Growing up brings a strange feeling, a bad premonition. There is too much homework, and I can’t finish it. What do you want from me? Just homework, what else can I do? Homework, ah, besides homework, what else is there? Ah, can’t we take a break from doing all this homework?*” This sentiment is worthy of adults’ reflection on the impact of their educational strategies on children’s enthusiasm for learning.

### 3.4. Relations

This theme highlighted how the children perceive the process of growing up through their social interactions with their significant figures, including parents, teachers, siblings, and classmates.

In terms of adult–child relations, the children perceived growing up as “*tinghua,*” which means showing respect, obedience, and compliance toward adults within Chinese contexts [51], akin to being “obedient” in English. As Dudu said, “*I used to be less tinghua when I was younger, now I am more tinghua.*” Observations in the classes indicated that the children learned to adhere to classroom rules and routines, participate in activities as directed by teachers, and display appropriate manners, such as greeting staff upon entering the preschool, maintaining quiet during class and lunch, rolling up their sleeves while washing hands, and avoiding whispering during naptime. Rewards like stickers or praise were given for exemplary behavior. Ningning, a boy, said, “*I got this sticker for playing with toys quietly. This one is for eating. I was second in eating that day, so I got it.*” At home, the children are expected to adhere to general behavioral requirements set by their parents, such as being polite, greeting others, eating well, putting away their toys and do more things independently. The children commented that as they grew, parental time decreased and rules set by parents became more numerous and stricter. This shift led to feelings of loss and a perceived distance in family relationships. As Momo expressed, “*It’s sad; when I was little, I could pull my brother’s ears and sleep with my mom. Now I sleep alone... When I was little, my brother used to tell me stories, but not anymore.*”

**Figure 2:**
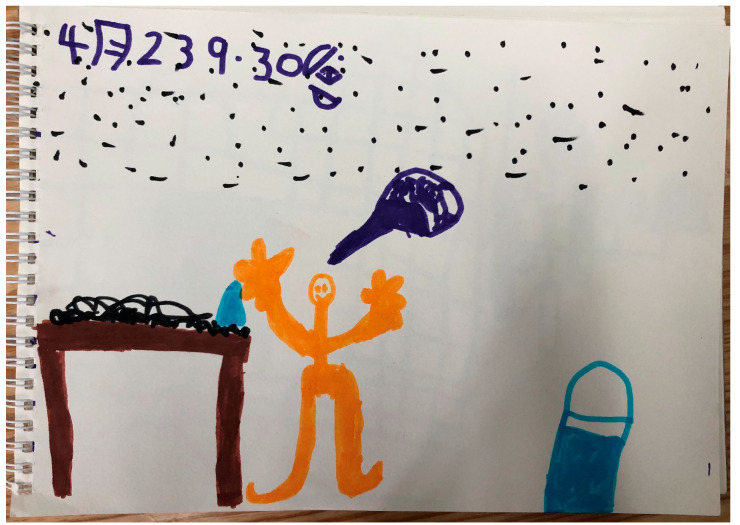
Chengcheng’s drawing: “I helped my granny wipe the table with water and held the bicycle for her [painted in the bubble]”.

The children also talked about how they gained a sense of growth from helping at home, even if it is only occasionally, such as mopping the floor, wiping tables, cooking, washing socks, serving meals, and receiving deliveries. As shown in Figure 2, Chengcheng drew how he helped his grandmother wipe the table and push the bicycle when her leg was injured. Manman, a girl, mentioned, “*I washed my dad’s socks, and they were stinky, so I had to wear a mask [while washing]. I help my mom find things because sometimes she doesn’t know where things fall, under the bed or on the sofa, so I help her find them. Today, my mom’s needle fell, and she asked me to help her find it... I can also cook by myself. My mom used a small knife to cut cucumbers for me; then, she helped me peel and beat the eggs. I stirred the eggs by myself, poured them [into the pan], and my mom added oil. Then, my mom helped me turn on the stove, and I stir-fried it myself. When my mom said ‘stop,’ I turned off the heat, and then a meal was ready.*”

Another category identified by the participating children concerned peer relations. Children from families with multiple offspring noted that growing up involves looking after their younger siblings. This dynamic is characterized more by shared playtime than by a caregiving role typical of adults [52], encompassing activities such as feeding them, playing with them, purchasing items for them with their allowances, and sharing rewards earned at preschool. Chechen, an older brother, reflected, “*I care for my younger sister. [Before] my relationship with my sister wasn’t good, and we often didn’t play together.*” Younger siblings mentioned the decrease in conflicts with their elder siblings as a part of their growing-up process. Haohao stated, “*I was naughty when I was little, and I would also make my sister angry. I don’t do that anymore because I’ve grown up.*” Moreover, establishing friendships in preschool is highlighted as a crucial element in fostering a sense of belonging among children [53,54] and an aspect of their growing up. Ranran, a boy, reported, “*Growing up feels great because I can make better friends. When I was little, I had only one friend, but now I have fifteen friends. My friends can play with me every day.*”

The children constructed their growth holistically and dynamically across four themes and twelve categories. These themes were rooted in the body, and became increasingly intertwined over time [10]. In the Theme Space, the physical development of the children enabled them to access more places. The grade levels in formal educational institutions (such as preschool and first grade) are correlated with their age in the children’s views [52] . In the Theme Skills, the gradual maturation of the central nervous system and motor skills provided physiological possibilities for the children to engage in various embodied projects. In the Theme Relations, infants are born with a primordial sense of connection with others based on physical perception, known as “syncretic sociability”, rather than purely cognitive operation [55,56] . Initially, infants closely depend on their primary caregivers, often the mother, while establishing relationships with other family members. Subsequently, they enter public spaces like the community, preschool, and extracurricular classes, expanding social interactions beyond the family.

## 4. Discussion

This study draws upon the structure–agency duality to explore how children develop their perceptions of growing up. This theoretical construct, pivotal in understanding the interplay between social structures (like norms and institutions) and human agency (or actions), enables a nuanced analysis of how children negotiate their identities within the confines of cultural expectations [57]. The ensuing sections will dissect this interplay, analyzing how the adults impart growth-related meanings and how the children, in turn, navigate and contribute to their own evolving stories of growing up.

### 4.1. Infusing Daily Situations with the Significance of Growing Up

This means that adults give the meaning of growing up to the unstructured, everyday situations that children are involved in. In these situations, as children interact and receive feedback from adults, they gain a sense of growing up, which further convinces them of the importance of these matters. In the Theme Body, they undergo a range of physiological transformations over time; however, from the voices of young children, it is evident that only certain changes are marked by adults as growing-up milestones, shaping how children view their lives. In Yueyue’s example, she recalled the experience of losing a tooth and the initial confusion and fear due to the unknown (it is not absolute; many children reported feeling nothing when their tooth fell out naturally). Her mother’s excitement and the belief that “losing teeth makes one grow up” helped Yueyue make the connection between losing teeth and growing up, thus alleviating her concerns during teething, and even making children look forward to losing teeth. Collective activities held by adults, such as birthdays, the Spring Festival, and the first day of school, allow children to directly experience the passage of time and age, helping them enter different areas of social life as members of a particular age group [52] . For example, Dudu said that she learned about the Chinese zodiac animals from a picture book library in a nearby mall, and her mother also discussed it with her. Her mother responded, “*When the Spring Festival comes, I will tell them it’s almost Chinese New Year, and you are another year older. At first, they didn’t understand, thinking that the date was January 1st. I would say, ‘No, that’s not the Spring Festival yet. We must wait a few more days until the lunar New Year.’ I would tell them what animal represents the current year and what animal will come next year. They would eagerly anticipate it, and as the zodiac sign changes, they would say, ‘Oh, this year, we will finish the Year of the Rat and start the Year of the Ox.’ Then they would ask me, ‘Mom, what is next year’s animal?’ and I would tell them.*”

In the Theme Relations, children feel they are growing up when they help with household chores. However, further interviews with parents revealed that most children only do so occasionally instead of persistently, and they may not necessarily perform the tasks well, often relying on caregivers’ assistance, as seen from Manman’s description of making scrambled eggs. Children remember doing chores because Chinese parents have always strongly emphasized family ethics. Therefore, when parents notice that children are lending a hand, they symbolically interpret this as children being able to take on some responsibility for the family. As a result, parents emotionally and even joyfully praise children for being sensible and grown-up, which evokes a sense of pride in children and fosters an acceptance of these cultural values.

### 4.2. Establishing Specific “Occupations” to Engage Children

Occupations refer to structured activities, whether educational or play-based, for preschool children rather than “people who help us” in this study. Froebel’s proposed the concept of occupations to enhance learning through play [58], including exploring clay, woodwork, cooking, parquetry, sewing, weaving, and painting. Dewey also named children’s socially purposeful activities in preschools as occupations [1]. Attending preschool, advancing to a higher class in the Theme Space, and learning to draw, play sports, and music in the Theme Skills are a series of occupations set for young children in modern society, also seen by children as their tasks for growing up. Children seem to believe they can only advance to a higher growth stage if they can accomplish these tasks; otherwise, they cannot grow up smoothly. In contemporary China, like in countries such as the UK and the US, most children aged 3 to 6 spend most of their waking hours in preschools [59]. They then enter primary school for further education. There are many levels in the educational continuum, and each time a child moves from one level to the other, a transition occurs. Researchers have investigated children’s understanding and experience of this turning point and the principles for successful transitions [47,60,61,62]. This paper further points out that children regard the upgrade in the educational space, that is, attending preschool and transitioning from *xiaoban* to *zhongban* and then to *daban,* as essential events in their growth. Moreover, the skills that the participating children mentioned often reflect the priorities and values of a specific society [52]. For example, the Guidelines for 3–6 Children’s Learning and Development of the Ministry of Education of the People’s Republic of China (2012) require children to jump rope and use spoons and chopsticks [63]. As a result, the children also mentioned these skills frequently [10]. There is an emphasis on academic education in Chinese culture. In contemporary society, education is seen as a tool for competing for limited social resources, leading to a pathological form called “Education-Anxiety” [64]. Parents shift academic pressure onto their children in preschool, making them take extracurricular academic courses when they enter *daban* [51]. However, the urban parents in the research not only focused on academic knowledge but also paid attention to their children’s artist talents and athletic skills, investing money and time to support them in learning to do sports, draw, dance, and so on. Therefore, the children viewed athletic, scholastic, and artistic skills as signs of growing up. 

### 4.3. Navigating Self-Constructed Growing up Narratives

Children play an active role in constructing their narratives and understandings of growing up [65,66]. From their narratives and the researcher’s observations, the children actively participate in social life affairs, explore different things, enjoy growth and progress, acquire new skills, behave in culturally appropriate ways, thus confirming their abilities, and feel pride, as Rogers believes individuals are endowed with an “actualizing tendency” [67]. Children also reshape societal influences and information in light of their life experiences and with a critical attitude. They engage in what Corsaro refers to as “interpretive reproduction” [68]. In the example of Yuyu, she began to critically consider and reflect on the messages her parents have conveyed to her, wondering whether “eating by oneself”, “weight gain”, and “height increase” truly represent growing up and which one is more persuasive among them all in determining a child is growing up or not. She depicted that physical enlargement is not a sign of growth, because if a baby is only a few months old but has a significant weight, we cannot say that the baby has grown much; we can only say that the baby has developed “*just a little bit.*” In the Theme Relations, *tinghua* is a significant virtue that Chinese culture hopes young children learn. It demands that children listen to their parents and teachers and comply with the customs and rules generally recognized by society to become moral people [8]. In the research, participants do not entirely suppress their inner thoughts and impulses and even internalize authority figures’ moral will, leading to false self-systems [69]. Still, instead, as in the case of Lulu’s complaint about her parents’ excessive supervision in the Theme Space, they yearn for freedom and power and want to control their lives rather than being restricted too much, even if she may not have openly resisted her parents face to face.

## 5. Conclusions

This study explores the detailed perspectives of young children on growing up. It reveals that children perceive their growth as an increasingly intricate engagement that embodies changes across four principal themes: body, space, skills, and relations. It points out that children develop their perceptions in a way characterized by “structure-agency”. As an exploratory study, it has some limitations. First, further in-depth exploration is required to understand how adults influence children’s views about growing up. Second, although children with differentiated performances that reflect the diversity of the classes were included as much as possible when choosing research participants, some biases may still exist, which need further exploration. Despite the limitations, the findings that preschoolers view growth as a dynamic and non-linear process complement the existing research in the developmental psychology and other fields, which often adopt an external perspective. From a practical standpoint, these findings can inform the design of more child-centered curricula and a more supportive environment. They can also be instrumental in guiding parents to better understand and support their children’s developmental needs. 

## Figures and Tables

**Table 1:** Themes, categories, and codes in the children’s voices.

Themes	Categories		Codes
Body	Physical changes	Physical enlargement	Grow taller, gain weight, limbs elongate, strength increase, faces enlarge, eyes get bigger, hair grows longer
		Physical loss	Lose teeth, diminish baby fat, decrease flexibility
	Age	\	Aging, have birthdays, have the Spring Festival, become old
Space	Educational space	\	Attend preschool, transition from *xiaoban* to *zhongban*, transition from *zhongban* to *daban*, start elementary school
	Accessible space	Expansion of space	Reach items in higher places, gain access to playground activities, leaving home to visit the shopping mall and preschool
		Restrictions of space	Cannot bounce on the sofa, cannot “steal” food in the kitchen, cannot go out to play until homework is finished
Skills	Daily living skills	Self-care skills	Walk, talk, dress, eat with a spoon or chopsticks, brush teeth and wash face, use the toilet and wipe independently
		Self-protection skills	Avoid playing with fire, avoid putting electric toys in the bathtub, avoid getting fingers caught in doors when opening or closing, avoid plugging and unplugging repeatedly, avoid touching power strips with wet hands
	Athletic skills	\	Jump rope, play basketball, run, somersaults, play soccer, play badminton, cycle (without training wheels), swim, ride a scooter, taekwondo, rollerblade, learn Chinese *kung fu*
	Artistic skills	Imitating	Create realistic drawings, produce abstract paintings, incorporate gradient colors, depict overlapping relationships, do splits, bend backward, play the piano (without music sheets), play the violin
		Expressing one’s creativity	Involve original ideas while painting
	Recreational skills	\	Engage in block building, play more complex games, learn to program
	Scholastic skills	Acquiring knowledge	Do plus and minus, recognize Chinese/English characters, learn Mandarin pinyin, learn Chinese idioms, write neatly
		Getting good grades	Score a perfect hundred, rank highly in extracurricular classes
Relations	Parent–child relations	*Tinghua*	Avoid using offensive language or lying, eat well, be polite, put away toys independently
		Helping at home	Wipe tables, sweep and mop the floor, cook noodles, stir-fry vegetables, serve meals, wash dishes, pick up a delivery
	Teacher–child relations	\	Listen to teachers quietly, sit straight in class, put away toys quickly when music is heard, keep quiet during meals, fold the quilt after nap time, receive praise, earn stickers
	Peer relations	Caring for siblings	Play with younger sibling, feed younger sibling, avoid disturbing older siblings, avoid damaging things that older sibling like
		Making friends	Make friends, make more friends

## Data Availability

The raw data supporting the conclusions of this article will be made available by the authors on request.

## Supplementary Materials

The following supporting information can be downloaded at https://www.mdpi.com/article/10.3390/bs14030253/s1, Table S1: Information about the participating children.
