# Supplementary material for: “Mom Said after the Spring Festival, I’ve Grown a Year”: Chinese Preschoolers’ Perspectives on Growing Up
Source: Behav Sci (Basel). 2024 Mar 20;14(3):253. doi: 10.3390/bs14030253 (PMC10967826; doi:10.3390/bs14030253)
Supplement: Supplementary file 1 [file behavsci-14-00253-s001.zip › behavsci-2888150-supplementary.pdf]

**SUPPLEMENTARY MATERIAL**

**Table S1** Information about the participating children

| Number | Pseudonym | Date of Birth | Gender | Paternal Education | Maternal Education | Primary Caregiver | Siblings and Age Difference | Institution Attended |
|---|---|---|---|---|---|---|---|---|
| C1 | Dudu | 2015.06 | Female | Bachelor's degree | Bachelor's degree | Mother | One sister, 3 years older | A Preschool |
| C2 | Momo | 2015.06 | Female | Master's degree | Bachelor's degree | Parents | One brother, 3 years older; one sister, 6 years younger | A Preschool |
| C3 | Yuyu | 2015.01 | Female | Bachelor's degree | Bachelor's degree | Parents, paternal grandmother | One sister, 4 years younger | A Preschool |
| C4 | Yaoyao | 2014.10 | Female | Bachelor's degree | Bachelor's degree | Parents, grandmothers | None | A Preschool |
| C5 | Manman | 2015.05 | Female | Bachelor's degree | Bachelor's degree | Parents, grandparents | One brother, 3 years older | A Preschool |
| C6 | Ziyue | 2015.04 | Female | Bachelor's degree | Bachelor's degree | Parents, maternal grandparents | None | A Preschool |
| C7 | Gege | 2014.10 | Female | Bachelor's degree | Bachelor's degree | Parents, paternal grandparents | None | A Preschool |
| C8 | Xiaoxue | 2015.01 | Female | Bachelor's degree | Bachelor's degree | Parents, paternal grandmother | One brother, 6 years younger | A Preschool |
| C9 | Lulu | 2015.01 | Female | Bachelor's degree | N/A | Parents | One older brother, age undisclosed | A Preschool |
| C10 | Qiqi | 2015.03 | Female | Bachelor's degree | Bachelor's degree | Parents | None | A Preschool |
| C11 | Xiaozao | 2015.04 | Female | N/A | Associate degree | Mother, maternal grandmother, a nanny | None | A Preschool |
| C12 | Zhuzhu | 2014.09 | Female | Bachelor's degree | Bachelor's degree | Parents | None | A Preschool |
| C13 | Xiaoquan | 2014.12 | Male | Bachelor's degree | Bachelor's degree | Mother, aunt | One sister, 6 years older | A Preschool |
| C14 | Ningning | 2015.04 | Male | Master’s degree | Bachelor's degree | Parents, paternal grandparents | None | A Preschool |
| C15 | Chenchen | 2014.11 | Male | Bachelor's degree | Bachelor's degree | Parents, great-aunt | One sister, 2 years younger | A Preschool |
| C16 | Chengcheng | 2015.01 | Male | Associate degree | Bachelor's degree | Parents, paternal grandmother | None | A Preschool |
| C17 | Qiaoqiao | 2014.09 | Male | Bachelor's degree | Bachelor's degree | Parents | One sister, 4 years older | A Preschool |
| C18 | Xuxu | 2015.07 | Male | Bachelor's degree | Bachelor's degree | Parents, paternal grandparents | None | A Preschool |
| C19 | Xiaocheng | 2015.05 | Male | Master's degree | Bachelor's degree | Parents, paternal grandparents | One brother, 3 years older | A Preschool |
| C20 | Hanghang | 2015.06 | Male | Doctoral degree | Bachelor's degree | Parents, paternal grandparents | One sister, 5 years younger | A Preschool |
| C21 | Maomao | 2015.05 | Male | Bachelor's degree | N/A | Parents, paternal grandparents | One older brother, age undisclosed | A Preschool |
| C22 | Yiyi | 2014.09 | Male | Bachelor's degree | Bachelor's degree | Parents, paternal grandparents | None | A Preschool |
| C23 | Xinxin | 2015.07 | Female | Technical school diploma | Associate degree | Parents | One sister, five years older | B Preschool |
| C24 | Tangbao | 2015.06 | Female | N/A | N/A | Parents | One older sister, age undisclosed | B Preschool |
| C25 | Xiaoyu | 2015.05 | Female | Bachelor's degree | Master’s degree | Parents, paternal grandmother | None | B Preschool |
| C26 | Feifei | 2014.10 | Female | Bachelor’s degree | Master’s degree | Mother, paternal grandparents | None | B Preschool |
| C27 | Miaomiao | 2015.06 | Female | Associate degree | Bachelor's degree | Parents, maternal grandparents | None | B Preschool |
| C28 | Yueyue | 2015.07 | Female | Associate degree | Bachelor's degree | Parents | One sister, 2 years younger | B Preschool |

| | | | | | | | | |
|---|---|---|---|---|---|---|---|---|
| C29 | Sining | 2014.10 | Female | Technical school diploma | Technical school diploma | Parents | None | B Preschool |
| C30 | Ningyi | 2015.01 | Female | Master’s degree | Bachelor’s degree | Parents, maternal grandmother | None | B Preschool |
| C31 | Jingyi | 2015.03 | Female | N/A | N/A | Parents, maternal grandmother | One older sister, age undisclosed | B Preschool |
| C32 | Yanyan | 2015.07 | Female | Associate degree | Associate degree | Parents, paternal grandparents | None | B Preschool |
| C33 | Xiaorui | 2015.08 | Male | High school diploma | N/A | Parents, maternal grandparents | None | B Preschool |
| C34 | Jiajia | 2014.10 | Male | Technical school diploma | Associate degree | Parents, grandmothers | One brother, 2 years older | B Preschool |
| C35 | Zhengzheng | 2014.12 | Male | Master’s degree | Master’s degree | Parents, maternal grandmother | None | B Preschool |
| C36 | Anan | 2015.08 | Male | Associate degree | Associate degree | Parents | One sister, 2 years younger | B Preschool |
| C37 | Xuanxuan | 2014.10 | Male | Junior high school diploma | Technical school diploma | Parents, maternal grandparents | One sister, 2 years older | B Preschool |
| C38 | Qiuqiu | 2015.04 | Male | Technical school diploma | Bachelor’s degree | Parents | None | B Preschool |
| C39 | Haohao | 2014.11 | Male | High school diploma | High school diploma | Parents | One sister, 8 years older | B Preschool |
| C40 | Ranran | 2015.02 | Male | Bachelor’s degree | Bachelor’s degree | Parents, maternal grandparents | None | B Preschool |
| C41 | Xiaobo | 2014.09 | Male | High school diploma | Junior high school diploma | Parents | One sister, 6 years older | B Preschool |
| C42 | Xiaoai | 2015.04 | Male | High school diploma | Junior high school diploma | Parents, paternal grandfather | One older sister, age undisclosed | B Preschool |
| C43 | Ruirui | 2016.08 | Female | Bachelor’s degree | Bachelor’s degree | Parents, maternal grandparents | One sister, 3 years older | C Preschool |
| C44 | Xiaoyou | 2016.06 | Female | Bachelor’s degree | Bachelor’s degree | Parents, paternal grandmother | One brother, 5 years older | C Preschool |
| C45 | Xiaomiao | 2015.10 | Female | Doctoral degree | Doctoral degree | Parents, maternal grandparents | None | C Preschool |
| C46 | Xiaoman | 2016.05 | Female | Bachelor's degree | Master's degree | Parents, paternal grandmother | None | C Preschool |
| C47 | Shishi | 2016.04 | Female | Bachelor's degree | Master's degree | Parents, maternal grandparents | None | C Preschool |
| C48 | Xixi | 2016.04 | Female | Bachelor's degree | Bachelor's degree | Parents, maternal grandmother | None | C Preschool |
| C49 | Xiaomo | 2016.04 | Female | Bachelor's degree | Master's degree | Parents, maternal grandparents | One brother, 2 years younger | C Preschool |
| C50 | Xiaoyuan | 2016.07 | Male | Master's degree | Bachelor's degree | Parents, paternal grandmother | None | C Preschool |
| C51 | Jiujiu | 2015.12 | Male | Master’s degree | Master’s degree | Parents, maternal grandparents | None | C Preschool |
| C52 | Dingding | 2015.10 | Male | Bachelor’s degree | Bachelor’s degree | Mother, maternal grandparents | One sister, 6 years older | C Preschool |
| C53 | Zhaozhao | 2016.05 | Male | Bachelor’s degree | Bachelor’s degree | Parents, paternal grandparents | One brother, 6 years older | C Preschool |
| C54 | Zimo | 2016.07 | Male | Master's degree | Master's degree | Parents, paternal grandmother | None | C Preschool |
| C55 | Hetao | 2015.10 | Male | Bachelor's degree | Bachelor's degree | Mother, maternal grandmother | None | C Preschool |
| C56 | Mumu | 2016.06 | Male | Bachelor's degree | Bachelor's degree | Parents, maternal grandmother | One sister, 3 years younger | C Preschool |

Note: The information in the table was provided by the parents and class teachers of the preschoolers. 'N/A' refers to the information that parents did not provide due to privacy concerns.
